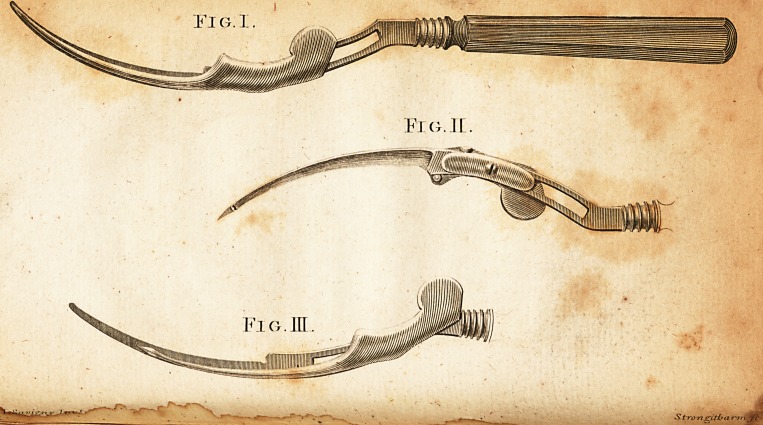# Description of an Improved Instrument for the Fistula in Ano

**Published:** 1790

**Authors:** J. Savigny

**Affiliations:** Surgical-Instrument Maker in London


II. *Description of an improved Instrument for the Fistula in Ano. Communicated in a Letter to Dr.* Simmons, *F. R. S. by Mr.* J. Savigny, *Surgical-Instrument Maker in London.*

IT has for a long time been a subject of complaint that the knife usually employed, by surgeons, in the operation for the fistula in ano, though very ingeniously contrived, and in most respects adequate to its intended purpose, is materially defective in one important particular, namely, the difficulty the operator experiences in forcing the button, at its extremity, through a diseased gut; a circumstance which must necessarily render the operation much more painful than it would otherwise be to the patient.

This complaint has induced me to direct my attention to the subject; and I flatter myself that the instrument I now beg leave, Sir, to submit to your consideration (and which has been honoured with the approbation of many eminent surgeons who have employed it in their practice) will be found to remedy the above-mentioned inconvenience.

It

It is compoſed of two blades (one fixed, the other moveable; the former with a cutting edge and blunt point, and the latter with an exquiſitely ſharp point, and blunt in all its other dimenſions) moſt accurately adjuſted to each other, and retained in a parallel poſition by a ſcrew and ſliding ſpring, which the annexed figures and the following references to them will more fully explain:

FIGURE 1. Repreſents the inſtrument in its proper ſtate to be introduced into the ſinus, with the ſliding blade ſo far advanced that its ſharp point is exactly on a level with and protected by the blunt one of the cutting blade.

FIG. 2. Gives it in another point of view, when it is ſuppoſed to be introduced as far as neceſſary, with the ſharp point protruded beyond the blunt one, to facilitate the paſſage of the button through the gut.

FIG. 3. Exhibits the ſliding blade withdrawn, after having conducted the button through its deſtination, with the edge of the cutting blade expoſed, and free from all impediment, to finiſh the operation.

*Pall Mall,*
July 2, 1790.

III. *An*

## Figures and Tables

**Fig. I. Fig. II. Fig. III. f1:**